# Successful treatment with bortezomib and dexamethasone for proliferative glomerulonephritis with monoclonal IgG deposits in multiple myeloma: a case report

**DOI:** 10.1186/s12882-017-0524-7

**Published:** 2017-04-06

**Authors:** Rio Noto, Nozomu Kamiura, Yuichiro Ono, Sumie Tabata, Shigeo Hara, Hideki Yokoi, Akihiro Yoshimoto, Motoko Yanagita

**Affiliations:** 1 Department of Clinical Nephrology, Kobe City Medical Center General Hospital, 2-1-1, Minatojimaminamimachi, Chuo-ku, Kobe-city, Hyogo 650-0047 Japan; 2 Department of Clinical Hematology, Kobe City Medical Center General Hospital, Hyogo, Japan; 3 Department of Diagnostic Pathology, Kobe University Hospital, Hyogo, Japan; 4 Department of Nephrology, Kyoto University Graduate School of Medicine, Kyoto, Japan

**Keywords:** Proliferative glomerulonephritis with monoclonal IgG deposits (PGNMID), Multiple myeloma, Monoclonal gammopathy

## Abstract

**Background:**

Proliferative glomerulonephritis with monoclonal IgG deposits (PGNMID) is a form of renal involvement by monoclonal IgG deposits that was found in mesangial, subendothelial or subepithelial regions. The distribution of glomerular deposits was completely different from that in monoclonal immunoglobulin deposition disease. PGNMID is reported to be rarely associated with a hematological malignancy. Previously, only five cases of PGNMID with multiple myeloma have been reported. However, the pathogenic relationship between PGNMID and multiple myeloma was unclear because a detailed description was not provided. We report that a patient with PGNMID associated with multiple myeloma was treated with bortezomib and dexamethasone and underwent the second renal biopsy after treatment, showing that chemotherapy was effective for PGNMID clinically and pathologically.

**Case presentation:**

A 75-year-old man presented with progressive leg edema, had nephrotic range proteinuria, hypoalbuminemia, moderate renal failure, and occult blood in his urine. Electrophoresis results showed serum and urinary monoclonal spikes of IgGκ type immunoglobulin. A renal biopsy specimen showed lobular mesangial proliferation with mesangiolysis, glomerular micro-aneurysm, and endocapillary hypercellularity. Immunofluorescence results revealed strong granular capillary and mesangial staining for IgG1, C3 and κ light chain in glomeruli without tubular deposits of any immunoglobulin. Electron microscopy also showed dense granular deposits in subendothelial and mesangial areas. PGNMID associated with multiple myeloma (IgGκ type) was diagnosed on the basis of a subsequent bone marrow examination. Bortezomib and dexamethasone therapy significantly reduced proteinuria and elevated serum albumin level. Eight months later, the second renal biopsy showed no active lesions and that the IgG1 and κ light chain deposits had drastically disappeared.

**Conclusions:**

This is the first case of PGNMID with multiple myeloma successfully treated with bortezomib and dexamethasone in which comparative renal biopsies were performed before and after treatment. Our findings suggest the pathogenesis of PGNMID and therapeutic options for PGNMID.

## Background

Nasr et al. recently described proliferative glomerulonephritis with monoclonal IgG deposits (PGNMID) as a novel form of glomerulonephritis that was positive for the deposition of a single IgG subclass and a single light chain isotype in mesangial, subendothelial, or subepithelial regions [1]. On electron microscopy (EM) analysis, these deposits were non-organized, granular, and similar to those found in monoclonal immunoglobulin deposition disease (MIDD). However, the distribution of glomerular deposits, which mimicked the deposits that occur with ordinary immune-complex glomerulonephritis, was completely different from that in MIDD [2].

The pathogenesis of PGNMID remains unknown. However, PGNMID recurrence in renal allograft patients suggests that this disease is caused by monoclonal immunoglobulin or other pathogenic factors circulating in the serum [3–5]. In 30% of PGNMID cases, there is a monoclonal component in serum [2]. However, PGNMID is rarely associated with a hematological malignancy [2]. Including our case, only six cases of PGNMID with multiple myeloma (MM) have been reported [2, 6–8]. In four previous cases, the pathogenic relationship between PGNMID and MM was unclear because a detailed description was not provided [2, 6, 7]. The other case with PGNMID complicated with MM was treated with bortezomib and dexamethasone (BD) therapy and her renal function improved after initiating treatment for MM, though they did not examine second renal biopsy after treatment [8].

Here, we describe a patient with PGNMID associated with MM, for which BD therapy improved PGNMID, comparing two renal biopsies obtained before and after chemotherapy for MM.

## Case presentation

A 75-year-old Japanese man with a history of hypertension, angina pectoris, and colon cancer presented at a local hospital with progressive fatigue. His laboratory test results showed serum albumin of 3.3 g/dL, hemoglobin of 8.0 g/dL, urine protein of 4+, and negative urinalysis for occult blood. Three months later, he presented with leg edema and loss of appetite and was referred to our hospital. He had nephrotic range proteinuria (6.0 g/g), hypoalbuminemia (albumin of 2.8 g/dL), an elevated serum creatinine level (1.1 mg/dL), and urinalysis showed 30-49 red blood cells per high-power field without cellular casts. Decreased levels of serum IgM (14 mg/dL) and IgA (60 mg/dL) compared with a normal IgG level (1202 mg/dL) suggested plasma cell dyscrasia.

**Fig. 1:**
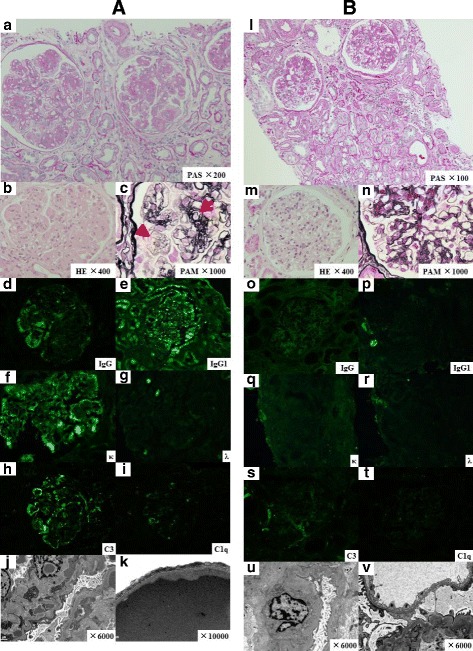
Renal biopsy histological features of PGNMID associated with multiple myeloma. First renal biopsy (**a**-**k**). **a** Periodic acid-Schiff (PAS) staining: renal histopathology showed lobular glomeruli with mesangial expansion and cell proliferation complicated with acute lesions, such as mesangiolysis, ×200. **b** Hematoxylin and eosin (HE) staining: glomerular micro-aneurysm and endocapillary hypercellularity, ×400. **c** Periodic acid methenamine silver (PAM) staining: duplication of glomerular basement membrane (an arrow) and mesangiolysis (an arrow head) was observed, ×1000. Congo red staining was negative (not shown). **d** Immunoglobulin G (IgG), **e** immunoglobulin G1 (IgG1), and **f** kappa immunostaining were detected on capillary walls and mesangial areas. (**g**) Lambda immunostaining was negative. **h** C3 immunofluorescence was positive (2+) on capillary walls, and (**i**) C1q was also weakly positive (1+) on some capillary walls. No positive immunofluorescence was found in tubular basement membranes. Staining for IgM and IgA was negative (not shown). IgG subclass analysis was positive for IgG1 only. IgG2, IgG3, and IgG4 were negative (not shown). EM (**j**) low-power and (**k**) high-power fields showed that electron-dense deposits in the subendothelial and mesangial areas exhibited a granular texture without a fibrillary appearance. Second renal biopsy (**l**-**v**). **l** Glomeruli showed less of an increase in mesangial matrix in the first renal biopsy and no mesangiolysis or micro-aneurysms with PAS staining, ×100. **m** A glomerulus showed no endocapillary hypercellularity with HE staining, ×400. **n** A glomerulus still had partial duplication of the glomerular basement membrane with PAM staining, ×1000. **o** IgG, (**p**) IgG1, (**q**) kappa, and (**r**) lambda immunofluorescence results were negative. (**s**) C3 immunofluorescence was weakly positive in a mesangial area. (**t**) No deposition of C1q. EM (**u**) low-power and (**v**) high-power fields showed no electron-dense deposits

Protein electrophoresis showed monoclonal spikes (IgGκ type) in both his serum and urine. A serum cryoglobulin test was negative. An abdominal ultrasound examination showed normal renal size and loss of corticomedullary differentiation in both kidneys. A renal biopsy specimen (Fig. 1A), which contained 24 glomeruli and 4 were sclerotic, showed lobular mesangial proliferation and expansion with mesangiolysis, glomerular micro-aneurysm, and endocapillary hypercellularity. The glomerular proliferative changes were global and almost all glomeruli showed similar pattern. Duplication of glomerular basement membrane was also observed. Congo red staining was negative. Tubulointerstitial scarring involved 30% of the renal cortex. No significant interstitial inflammation was observed. Arteriosclerosis was severe.

In the immunofluorescence studies, the manufactures and dilutions of antibodies were as follows: IgG (MP Biomedicals, Santa Ana, CA, USA; dilution 1:400), IgA (MP Biomedicals; dilution 1:200), IgM (MP Biomedicals; dilution 1:100), C3 (MP Biomedicals; dilution 1:200), C1q (MP Biomedicals; dilution 1:50), IgG1, 2, 3, and 4 (Thermo Fisher Scientific, Waltham, MA, USA; dilution 1:200), κ and λ (SouthernBiotech, Birmingham, AL, USA; dilution 1:50). Results showed glomeruli with granular capillary and mesangial staining for IgG (2+), C1q (1+), and C3 (2+) and light chain isotype restriction limited to κ; however, there were no tubular deposits. Staining for IgG subclasses was positive for IgG1 only, and negative for IgG2, IgG3, and IgG4. Electron-dense deposits in the subendothelial and mesangial areas exhibited granular texture and did not have fibrillary appearance. A bone marrow examination revealed 17% IgGκ-positive monoclonal plasma cells and confirmed a diagnosis of PGNMID associated with MM (IgGκ type).

**Fig. 2:**
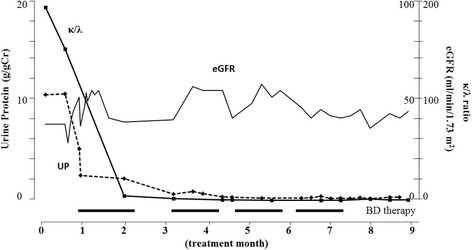
Clinical course. After initiating prednisolone therapy, this patient underwent four cycles of BD therapy (bortezomib at 1.3 mg/m^2^/week, and dexamethasone at 20 mg/week), followed by bortezomib maintenance therapy (1.3 mg/m^2^, biweekly). The κ/λ ratio, urinary protein (UP), and eGFR (calculated using equations for Japanese [26]) results are shown over the course of treatment

After his first kidney biopsy but before his diagnosis of MM, this patient received methyl-prednisolone pulse therapy for 2 days. After his diagnosis of MM, he was started on weekly BD therapy (bortezomib at 1.3 mg/m^2^/week, dexamethasone at 20 mg/week, 4-week treatment per cycle). Prior to initiating BD therapy, his serum albumin was 2.7 g/dL, creatinine was 1.52 mg/dL, κ/λ ratio was >186, and urinalysis showed 12.3 g/g of protein and 2+ occult blood. After the first BD course, laboratory test results improved remarkably, with serum albumin of 3.2 g/dL, creatinine of 1.42 mg/dL, κ/λ ratio of 5.09, and urine protein of 2.16 g/g. After the fourth course of BD therapy, serum albumin was 3.6 g/dL, creatinine was 1.00 mg/dL, κ/λ ratio was 1.10, and urinalysis showed 0.25 g/g protein without occult blood (Fig. 2). This patient underwent four cycles of BD therapy followed by bortezomib maintenance therapy (1.3 mg/m^2^, biweekly). No adverse effects were observed and the laboratory results were stable.

A second renal biopsy was performed 8 months after initiating BD therapy (Fig. 1B). The biopsy specimen contained 26 glomeruli and 6 were sclerosed. Although renal biopsy results still showed the duplication of glomerular basement membrane, mesangial matrix expansion, and weak deposition of C3 in mesangial areas, there were no active lesions of mesangiolysis, glomerular micro-aneurysm, and endocapillary hypercellularity and no deposits of IgG and κ light chain. The tubulointerstitial scarring was observed in 30% of the renal cortex. There was no significant interstitial inflammation. Arteriosclerosis was severe. An EM examination also showed no electron-dense deposits in a glomerulus. Our findings showed that BD therapy had eliminated the pathogenic monoclonal protein in his serum and had reversed the renal damage associated with PGNMID.

## Discussion

This report describes the case of a 75-year-old man with nephrotic syndrome, who was diagnosed with PGNMID associated with IgGκ type MM and successfully treated with BD therapy. In a renal biopsy after this treatment, active lesions and IgGκ deposits in the kidney had disappeared. PGNMID had remitted both clinically and pathologically after serum IgGκ monoclonal proteins had disappeared. Taken together, our findings suggest that MM had caused this patient’s PGNMID.

In a case series of PGNMID reported by Nasr et al., only 1 of 37 had MM for several years and received stem cell transplantation (SCT), although the details were not reported [2]. Guiard et al. reported two cases of non-cryoglobulinemic glomerulonephritis with monoclonal immunoglobulin deposits complicated with MM [6]. One patient was finally treated with thalidomide and corticosteroid, who developed end-stage renal disease. One was diagnosed as having MM 81 months after renal presentation, which may suggest that multiple myeloma had not evoked PGNMID. Redondo-Pachón et al. reported the details of a case of PGNMID with MM that was refractory to treatment [7]. In that case, the types of immunoglobulin in the myeloma cells and kidneys were not identical, suggesting that the PGNMID was not related to MM. Recently, Watanabe et al. have shown that a patient with PGNMID complicated with MM was treated with BD therapy and achieved a partial response with decreased serum monoclonal protein and improved renal function [8]. Although they did not examine renal histology after treatment, immunofluorescence with IgG subclasses and electron microscopic analysis of a glomerulus, the response for the BD therapy was consistent with our case that the patient reduced proteinuria and elevated serum albumin level after the first BD course.

Monoclonal gammopathy of undetermined significance (MGUS) is a condition characterized by the presence of monoclonal gammopathy without end organ damage [9]. There are two ways by which MGUS transitions to a malignant and symptomatic condition: (1) somatic genetic abnormalities occur in tumor cells, which results in MM; and (2) the clone produces a protein with an altered conformation, which causes progressive organ dysfunction and conditions like light-chain amyloidosis, MIDD, and cryoglobulinemia [10].

For patients with MM, 12-30% developed primary amyloidosis, although the treatment of primary amyloidosis was difficult at the time of melphalan and prednisone therapy [11, 12]. Only a minority of patients treated with this regimen responded and their median survival was 12 to 18 months [13, 14]. This therapy had remained unchanged for two decades until SCT was introduced. After SCT with high-dose melphalan was found to be effective for MM, the same regimen was also used for primary amyloidosis. A recent report stated that the median survival time was 4.6 years for patients treated with SCT and high-dose melphalan [15]. Using chemotherapy for myeloma during the management of primary amyloidosis is logical, as it may rapidly eradicate amyloidogenic light chains that are produced by a clonal plasma cell population; this is also true for treating MIDD [16–18].

Guiard et al. used rituximab to treat non-cryoglobulinemic glomerulonephritis with monoclonal immunoglobulin deposits and without a hematologic malignancy [6]. Rituximab was used in combination with other chemotherapy regimens for two patients with low-grade lymphoma and alone for five patients with no malignancy. In five patients, complete remission of nephrotic syndrome was achieved. The excellent results obtained with rituximab therapy are very encouraging as compared with the results obtained using corticosteroids alone or alkylating agent-based regimens; this suggests that B-cell regulation can ameliorate renal changes in PGNMID.

All patients who present with a well-defined severe hematologic malignancy, such as MM or high-grade non-Hodgkin lymphoma, must be treated according to standard chemotherapy protocols, which often include newly introduced drugs, such as thalidomide, bortezomib, and rituximab [19–21]. If a treatment achieves a hematologic remission and suppresses circulating monoclonal immunoglobulins, then the renal complications caused by a hematologic malignancy, including PGNMID, may disappear, as was observed in our case. In contrast, plasma cell dyscrasia or lymphoproliferative disorders can be involved in the pathogenesis of PGNMID with monoclonal gammopathy, similar to their involvement in MIDD and light chain amyloidosis. As Guiard et al. demonstrated [6], a specific treatment for MM or lymphoma, such as with bortezomib and rituximab, may also be an effective treatment for PGNMID without an overt malignancy [22].

There is still debate about the desirable strategy of the second biopsy in kidney diseases, especially in lupus nephritis [23–25]. In the renal diseases related to monoclonal immunoglobulins, however, there has been no report discussing the clinical strategy of repeat biopsy. In the present case, the 2nd biopsy pathologically showed amendment in active lesions such as endocapillary hypercellularity and mesangiolysis, reflecting the amendable effect of bortezomib and dexamethasone on histological damages triggered by monoclonal immunoglobulins. Therefore, the current case provides the first evidence that monoclonal immunoglobulin-associated PGNMID can be cured pathologically by eliminating the abnormal clonal proliferation of B-cells or plasma cells. To circumvent the potential risk related to the biopsy procedure, the improved histological features can be inferred from the improved laboratory test including serum albumin, creatinine, κ/λ ratio and urine protein.

## Conclusions

We have described a patient with PGNMID that was associated with MM and who was successfully treated with BD therapy, which sheds some light for unraveling the mechanism underlying PGNMID. Further studies are necessary to determine whether PGNMID, particularly with monoclonal gammopathy suggestive of plasma cell dyscrasia, should be treated with the specific chemotherapy used to treat MM.

## Abbreviations

BD: Bortezomib and dexamethasone
eGFR: Estimated glomerular filtration rate
UP: Urinary protein
